# Cell Membrane-Derived Vesicle: A Novel Vehicle for Cancer Immunotherapy

**DOI:** 10.3389/fimmu.2022.923598

**Published:** 2022-07-07

**Authors:** Caili Xu, Dianwen Ju, Xuyao Zhang

**Affiliations:** Department of Biological Medicines & Shanghai Engineering Research Center of Immunotherapeutics, School of Pharmacy, Fudan University, Shanghai, China

**Keywords:** cellular vesicle, drug delivery vehicle, cancer immunotherapy, combination therapy, membrane hybridization, drug encapsulation

## Abstract

As nano-sized materials prepared by isolating, disrupting and extruding cell membranes, cellular vesicles are emerging as a novel vehicle for immunotherapeutic drugs to activate antitumor immunity. Cell membrane-derived vesicles inherit the surface characteristics and functional properties of parental cells, thus having superior biocompatibility, low immunogenicity and long circulation. Moreover, the potent antitumor effect of cellular vesicles can be achieved through surface modification, genetic engineering, hybridization, drug encapsulation, and exogenous stimulation. The capacity of cellular vesicles to combine drugs of different compositions and functions in physical space provides a promising vehicle for combinational immunotherapy of cancer. In this review, the latest advances in cellular vesicles as vehicles for combinational cancer immunotherapy are systematically summarized with focuses on manufacturing processes, cell sources, therapeutic strategies and applications, providing an insight into the potential and existing challenges of using cellular vesicles for cancer immunotherapy.

## Introduction

Immunotherapy brings great hope to cancer patients, but it also faces challenges such as low response rate, difficulty in eradication, and susceptibility to relapse. Combination therapy offers promising solutions to address these issues (1). The appropriate delivery system physically combines multiple drugs, providing an integrated solution to achieve tumor targeting, killing, and activation of immune systems simultaneously. The main problem with conventional drug delivery systems such as liposomes, polymer micelles, dendrimers and nanogels is their vulnerability to clearance by the reticuloendothelial system and other circulating immune cells, resulting in severe liver toxicity and inadequate enrichment in target sites (2). With this in mind, researchers focus on the study of biomimetic drug vehicles (3, 4). Cellular vesicle is an important area of interest within the field of biomimetic drug delivery vehicles. It mainly refers to plasma membrane structures extracted from parental cells under external intervention and prepared into nano-sized vesicles for drug delivery (5).

In this review, we seek to track the recent advances in the application of cell membrane vesicles as drug vehicles for cancer immunotherapy. We introduce the manufacturing workflow of cellular vesicles and summarize their characteristics from various parental origins. Then, the currently reported strategies of utilizing cellular vesicles to combat tumors are comprehensively reviewed. And finally, the comparion with other nanovehicles and challenges of cellular vesicles in cancer immunotherapy are discussed in depth with the aim of accelerating the clinical applications of this novel platform for cancer immunotherapy.

## Manufacturing of Cellular Vesicles for Cancer Immunotherapy

### Isolation

**Figure 1:**
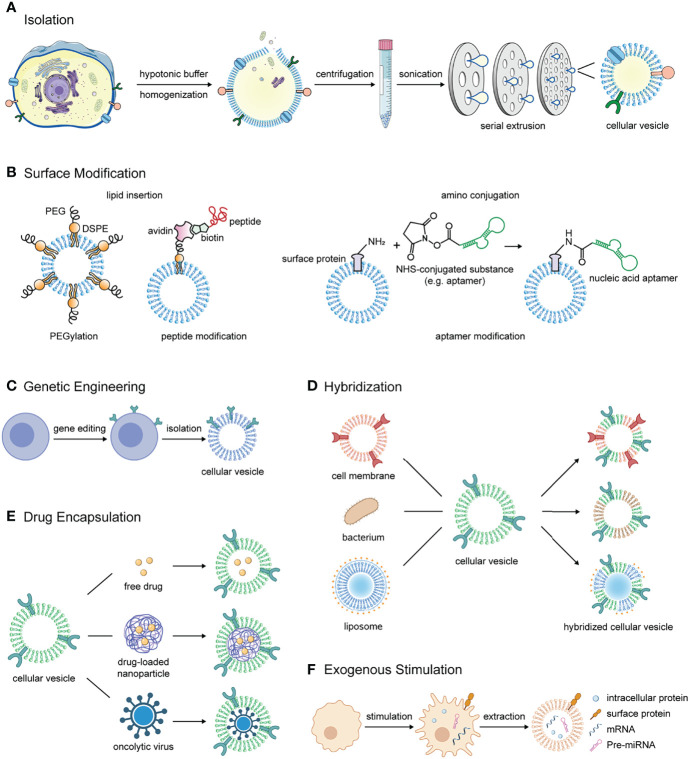
The manufacturing process, modification methods, and application strategies of cellular vesicles in combinational immunotherapy of cancer. **(A)** The process of isolating cell membrane-derived vesicles from parental cells. **(B)** Methods for modifying substances such as polyethylene glycol, tumor targeting peptide and nucleic acid aptamer on the surface of cellular vesicles. **(C)** Cellular vesicles inherit antitumor proteins that the parental cells overexpress through gene editing. **(D)** Cellular vesicles are hybridized with different materials such as cell membranes from other sources, bacterial membranes and liposomes to obtain multiple components and functions for cancer immunotherapy. **(E)** Free drugs, drug-loaded nanoparticles or oncolytic viruses are encapsulated in cellular vesicles for delivery to the tumor lesions to activate antitumor immunity. **(F)** In response to exogenous stimulation, immune cells produce a variety of tumor growth inhibitors including surface markers, intracellular proteins, mRNA for pro-inflammatory cytokines, and certain miRNAs, which can be retained in cellular vesicles.

The typical process of isolating cellular vesicles consists of several steps (**Figure 1A**). Firstly, the parental cells are harvested and resuspended in a hypotonic buffer, rendering the cytoplasm swollen and susceptible to fragmentation by external forces (6, 7). Then, if the cytoplasmic components are to be removed, cells usually need to undergo approximately five freeze-thawing cycles and dounce homogenization to release intracellular proteins (6, 7). Next, cell membranes are separated from other cellular components by continuous high-speed or density gradient centrifugation. For example, the cells were subjected to centrifugation at 1000 g, 10000 g, and 100000 g to remove the nuclei, organelles, and other impurities, respectively (6). For density gradient centrifugation, cell membranes were prepared by centrifugation through discontinuous 30-40-55% sucrose (w/v) density gradient. At the interface of the different sucrose solutions, three lipid rings could be clearly detected, where the fraction between 30% and 40% sucrose retained the most plasma membrane proteins and was then collected and prepared for vesicles (7–9). Finally, they are sonicated for several minutes and repeatedly extruded through about three layers of polycarbonate membranes with stepwise decreasing pore size to obtain cellular vesicles (6, 10). Besides, to obtain vesicles that retain cytoplastic proteins and RNAs, cellular vesicles can be purified by OptiPrep density gradient centrifugation. Briefly, the cells underwent serial extrusion of the polycarbonate membranes, followed by centrifugation at 100000 g through 10% and 50% OptiPrep medium, and vesicles were harvested at the junction of the two layers (11–14). Aside from the above typical procedures, cellular vesicles can be induced by cytochalasin B or obtained by nitrogen cavitation (15–17).

### Surface Modification

Surface modification of cellular vesicles is a vital strategy to improve their stability and tumor targeting ability. The modification methods include lipid insertion and amino conjugation. In terms of modification contents, they mainly include peptides, nucleic acid aptamers, and polyethylene glycol (PEG) (**Figure 1B**).

Attachment of tumor-targeting peptides is one of the most commonly used modification. One approach is to resort to lipid insertion and biotin-avidin interaction (18). Another approach is conjugation *via* N-hydroxysuccinimide (NHS) group. NHS esters are capable of covalent coupling with primary amines on proteins. Therefore, NHS-PEG-folic acid could be linked with amino groups of vesicle proteins for targeting tumors highly expressing folate receptors (19, 20). The modification of nucleic acid aptamers can also be performed by amino coupling to achieve specific targeting of tumors with high expressions of nucleolin (15). PEGylation is able to increase the dispersion stability and prolong the circulation time (21–23).

## Potential of Cellular Vesicles From Diverse Parental Cells in Cancer Immunotherapy

### Tumor Cells

Tumor cell membrane-derived vesicles have some unique properties in cancer therapy including persistent existence, homotypic targeting, and antigen stimulation. Tumor cells can evade clearance by macrophages through expressing innate immune checkpoint CD47 to convey a negative signal of phagocytosis (24). This property was well preserved on its cellular vesicles and enabled a prolonged circulation *in vivo*. However, other immunosuppressive molecules expressed on tumor cells such as PD-L1, Galectin-9 and Siglec-15 may also be retained on cellular vesicles and inhibit the function of tumor-infiltrating lymphocytes (25–27).

Another important rationale for employing cancer cell vesicles as antitumor drug vehicles is homotypic targeting, which is probably through surface adhesion molecules such as N-cadherin and galectin-3 (28, 29). In a tumor self-targeting study, researchers prepared four kinds of cell membrane-encapsulated magnetic nanoparticles derived from different tumor cell lines. In the *in vivo* competition of tumor “homing”, vesicles derived from heterologous tumor cells were notably weaker than that from homologous cells (28). This finding was also verified in patient-derived xenograft (PDX) models. The fluorescence intensity of tumor cell membrane-encapsulated nanoparticles at the tumor site was 3 and 10 times higher than that of erythrocyte membrane-coated nanoparticles and bare nanoparticles, respectively. For tumor cell vesicles derived from various patients, 2.5 to 10-fold higher tumor-targeting capacity was observed when the source of the donor membrane was consistent with the host compared to inconsistent cases (30).

Carrying tumor antigens is also a non-negligible advantage of tumor cell-derived cellular vesicles. Tumor cell vesicles inherit tumor-associated antigens and tumor-specific antigens and can therefore be equipped with immunological adjuvant for the preparation of cancer vaccine (21, 31–33).

### Immune Cells

The advantages of selecting immune cells as the source of cellular vesicles for antitumor drug vehicles are ease of genetic modification, natural cargo of antitumor components, and the ability to evade immune surveillance, target tumor cells and present tumor antigens.

Abnormal nuclear structure in erythrocytes and platelets as well as excessively active DNA replication and mutation in tumor cells pose additional impediments to gene editing. As for the immune cells, genetic engineering technologies have been widely used in manufacturing immune cells with chimeric antigen receptors (CARs) (34–37). Coating IR780-loaded mesoporous silica nanoparticles with GPC3-specific CAR-T cell membranes enhanced tumor-targeting capability, with tumors weighing less than half the weight of normal T cell membrane-coated nanoparticle treatment group (38).

Another noteworthy point is that vesicles extruded from immune cells carry naturally expressed pro-inflammatory and antitumor substances (13). Programmed cell death-1 (PD-1) and transforming growth factor-beta receptor (TGF-βR) expressed on T cells are considered to inhibit the antitumor effects of CD8^+^ T cells, however their retention on T-cell vesicles in turn neutralizes PD-L1 and TGF-β in the tumor microenvironment (TME). Meanwhile, antitumor substances expressed by T cells such as granzyme B and FasL can still induce apoptosis of tumor cells through their vesicles (13, 39). Studies on macrophages showed that cellular vesicles derived from M1-type macrophages contained high levels of IL-6 and tumor necrosis factor-alpha (TNF-α), which presented pro-inflammatory and tumoricidal effects *in vivo* (11, 14). Intravenous injection of M1 macrophage-derived vesicles alone was demonstrated to promote tumor-associated macrophage polarization toward M1 type and improve CD8^+^ T cell infiltration in TME (14).

In addition, vesicles originating from certain immune cells can evade clearance, target tumor sites, recognize cancer cells and present tumor antigens. The use of monocyte- and macrophage-derived vesicles as nanovehicles emphasizes on their ability to evade clearance by the mononuclear phagocyte system and the expression of α4β1 that interacts with the vascular cell adhesion molecule-1 (VCAM-1) of metastatic tumors (8, 40, 41). It was reported that macrophage J774 membrane-encapsulated nanoparticles exhibited delayed liver accumulation, with integrity maintained for up to 40 min, which is 2-fold longer than that of naked nanoparticles. Also, 25% of adherent particles coated with macrophage membrane were not phagocytized by Kupffer cells, significantly higher than uncoated nanoparticles (~ 9%). Drug delivery with macrophage vesicles increased the particle density at the tumor sites by approximately twofold (8). Besides, T-cell vesicles were shown to carry high levels of lymphocyte function-associated antigen-1 (LFA-1), which mediates the targeting of tumor sites *via* binding to intercellular adhesion molecule-1 (ICAM-1) highly expressed on tumor cells and inflamed endothelium (8, 39). Natural killer (NK) cell vesicles also have some degree of tumor homing ability due to aberrant expression of ligands for NK cell receptors (e.g., NKG2-D) in tumors (9). Neutrophil-derived vesicles have been shown to target tumor cells and inflammatory endothelium through three pairs of interactions including LFA-1/ICAM-1, β1 integrin/VCAM-1, and CD44/L-selectin (42). And DC vesicles could present tumor antigens to activate T cells (43, 44).

### Erythrocytes and Platelets

The abundance in quantity and simplicity in composition have led to the extensive use of erythrocyte membranes as drug vehicles (45). Similar to tumor cells, erythrocytes also highly express CD47 to protect themselves from the attack of macrophages, thus prolong the circulation time (46–48).

Platelets play a significant role in tumor metastasis (49). In analogy to erythrocytes, platelets regulate self-homeostasis in the circulation by expressing phagocytic negative signal CD47 (50). Besides, it is worth noting that platelet-derived vesicles contain P-selectin protein. CD44 is highly expressed and acts as the primary P-selectin ligand on certain types of carcinoma cells. Therefore platelet-derived vesicles probably have some degree of tumor-targeting ability (51–53). However, it was also reported that platelet-derived vesicles rapidly bound to blood monocytes due to P-selectin-mediated adhesion, which would reduce their stability and half-life (54).

### Other Types of Cells

In addition to the cells aforementioned, cellular vesicles obtained from fibroblasts and bacterial membrane for cancer treatment have also been reported. Fibroblast membrane-derived vesicles are superior in penetration into the TME, and bacterial membranes contribute to activation of the innate immune system (55–57).

## Strategies and Applications of Cellular Vesicles in Combinational Immunotherapy of Cancer

### Genetic Engineering of Cellular Vesicles

Gene editing allows cells to express proteins targeting tumor lesions or substances regulating immunity of TME, and the functionality can be perpetuated to their extruded vesicles (**Figure 1C**). For example, lentivirus carrying sequence of signal regulatory protein alpha (SIRPα), a ligand of CD47, and plasmid inserted with PD-1 sequence were transfected into different tumor cells. After being prepared into cellular vesicles separately, they were fabricated into fusion vesicles. The fusion vesicles were demonstrated to possess high levels of both SIRPα and PD-1 on the surface and have the ability to block innate and adaptive immune checkpoints simultaneously (10). To achieve targeted killing of tumor cells, CAR-T cells were extruded into cellular vesicles for hepatocellular carcinoma treatment (38). Besides, cellular vesicles were also engineered to overexpress antigens to stimulate T cell activation (58).

### Hybridization of Cellular Vesicles From Different Type of Cells or With Other Materials

Hybridized cellular vesicles inherit function characteristics of both parental materials (**Figure 1D**). Fused or hybridized cellular vesicles can be produced by breaking up, mixing and then co-extruding through polycarbonate porous membranes. A large number of works have investigated the fusion of two or even more types of cell membrane vesicles to achieve multi-functionality (10, 14, 29, 59).

Bacteria are one of the most common immune stimulants, and Escherichia coli (*E. coli*) membrane vesicles have been successfully employed to transport tumor antigens and act as vaccines in the absence of adjuvants (60, 61). Researchers constructed fusion vesicles by hybridizing *E. coli* outer membrane vesicles and tumor cell vesicles in order to simultaneously augment innate and adaptive immunity for personalized tumor immunotherapy. The fusion vesicles could be effectively enriched in lymph nodes and inhibited the growth and lung metastasis of colorectal and breast tumors (57). In another similar study, tumor cell membrane and *E. coli* cytomembrane were co-extruded with nanoparticles to obtain hybrid vaccine. In 4T1 tumor model, the tumor-suppressive effect of fusion vesicles was significantly better than the simple combinational dosing of tumor vesicle-nanoparticles and *E. coli* vesicle-nanoparticles, with 60-day survival rates improved from 0 to 93% and 25%, respectively (62).

In addition, the advantages of liposomes can also be conferred to cellular vesicles by hybridization (9, 41). For instance, decoration of emtansine-carrying liposomes with macrophage membranes displayed a significant suppressing effect on lung metastasis of breast cancer (41).

### Entrapping Drugs in the Hollow Cores of Cellular Vesicles

The hollow-core structure of cellular vesicles provides space for loading antitumor drugs and entrapping nanoparticles (**Figure 1E**). Encapsulation of free therapeutic agents can be achieved by co-incubation or remote loading (12, 63). And simple mixing of cellular vesicles and nanoparticles followed by co-extrusion is sufficient to prepare the desired cell membrane-coated nanoparticles (64). However, the surface charge interaction needs to be taken into account before wrapping the nanoparticles (65, 66). Studies of cellular vesicles loading chemotherapeutic drugs such as docetaxel, doxorubicin, camptothecin and oxaliplatin have been extensively reported (9, 18, 43, 48). These drugs can induce immunogenic cell death, and the combination with cellular vesicles confers an enhanced immune response. One of the interesting attempts was the combination of mature DC vesicles with oxaliplatin-loaded nanoparticles. Chemotherapy led to immunogenic cell death, and the camouflaged mature dendrosomes initiated T cell responses by presenting tumor antigens, thus amplifying antitumor immune responses (43). Cellular vesicles were also widely applied in cancer photothermal therapy, such as membrane-camouflaged indocyanine green nanoparticles, black phosphorus quantum dot and melanin nanoparticles (20, 38, 67–69). Oncolytic viruses can also be enveloped in cellular vesicles aiming to evade antiviral neutralizing antibodies and enhance tumor-targeting ability (70).

### Stimulating Production of Natural Antitumor Substances Before Vesicle Extraction

Stimulating immune cells to express endogenous antitumor substances and then extruding for vesicles is also an important strategy (**Figure 1F**). Vesicles isolated from activated or polarized immune cells were able to retain not only the membrane protein properties of the parental cells but also their intracellular proteins, mRNA and miRNA under appropriate preparation (11, 13, 14). It was reported that M1-type macrophages-derived nanovesicles contained high levels of mRNA of multiple pro-inflammatory cytokines and they could promote macrophage polarization toward M1 and infiltration of CD8^+^ T cells into tumors (11, 14).

## Comparison of Cellular Vesicles With Other Nanovehicles

The advantages of non-biomimetic nanovehicles (e.g., liposomes, polymer micelles, dendrimer, nanogels, mesoporous silica, metallic nanoparticles) include high yield, diverse chemical modifications and precise regulation of physicochemical properties. For example, pH-sensitive dendrimers, temperature-responsive nanogels and magnetic nanoparticles enable specific drug release, size switching, sol-gel transition, and magnetic hyperthermia at tumor sites (71–74). In comparison with that, cellular vesicles preserved membrane characteristics and functions of parental cells, thus exhibiting better biocompatibility, low immunogenicity, negligible toxicity, long circulation, and natural targeting ability (75).

Exosome is also emerging as an important drug delivery platform for cancer immunotherapy. Compared to cell membrane vesicles, the difference between exosomal surface proteins and cytoplasmic membranes offer unique possibilities for exosome as drug vehicles. For instance, co-expression of peptides with proteins highly expressed on exosome surface (e.g., tetraspanin CD9/CD63/CD81, LAMP-2B and lactadherin) by genetic engineering allows for their enrichment on exosomes (76–81). Anchoring drugs to the exosomal marker *via* a medium such as CP05 peptide-mediated CD63 linkage simplifies drug loading approaches (82). However, for cancer therapy, exosomes are mostly administered at doses of 100-600 μg exosomal proteins per mouse, which implies consumption of approximately 1 L cell supernatant, severely hindering the application of exosomes as drug vehicles (83, 84). Cell membrane vesicles and exosomes share most of the characteristics of biomimetic nanovehicles, yet the former has superiority in terms of yield, production stability, and size homogeneity (11, 12). Cell membrane vesicles are administered at doses similar to exosomes, but with yields up to 30-300 μg vesicle proteins/10^7^ cells (12, 17, 41).

## Future Perspectives

Through genetic modifications, membrane hybridization, drug encapsulation and exogenous stimulation, cellular vesicles were engineered to provide ideal vehicles for cancer immunotherapy drugs, including not only surface proteins but also internal nanoparticles, proteins, nucleic acids, and small-molecule drugs (**Table 1**).

**Table 1:** Applications of cellular vesicles in cancer immunotherapy.

Strategies	Intervention	Parental cell	Mechanisms	Tumor models	References
**Genetic engineering**	CAR-T cell vesicle-coated nanoparticle	T cell	GPC3-specific CAR-T membrane vesicles were used to wrap IR780-loaded mesoporous silica nanoparticles for tumor targeting and photothermal therapy.	Xenograft model of human liver cancer.	(38)
	SIRPα and PD-1	Tumor cell	Tumor cells were programmed to overexpress SIRPα and PD-1 and then extracted for cellular vesicles to simultaneously block innate and adaptive immune checkpoints *in vivo*.	Breast cancer and melanoma models. Recurrence and metastasis model of breast cancer.	(10)
**Membrane hybridizaiton**	Various cell membranes	Two types of tumor cells; Macrophage, platelet and tumor cell	Hybridization of two or more types of cellular vesicles from tumor cells, erythrocytes, platelets and immune cells to achieve the multiple functions of escaping clearance, targeting tumor leison and activating antitumor immunity.	Primary, recurrence and metastasis tumor model of breast cancer and melanoma.	(10, 14)
	Cell membrane and bacterial membrane	Tumor cell and bacterium	Tumor cell vesicles were fused with *E. coli* membrane vesicles to stimulate dendritic cell maturation and T cell activation for personalized cancer vaccines and immunotherapy.	Breast and colon cancer models. Lung metastasis model of breast cancer.	(56, 62)
	Cell membrane and drug-loaded liposome	Macrophage; Natural killer cell	Liposomes carrying antitumor drugs (emtansine or doxorubicin) were hybridized with macrophage or NK cell vesicles for targeted cancer therapy through interactions of α4β1/VCAM-1 and NKG2-D and its ligands, respectively.	Lung metastasis model of breast cancer. Xenograft tumor model of human cancer cells.	(9, 41)
**Drug encapsulation**	DC vesicles, oxaliplatin-loaded nanoparticles and αPD-L1	Dendritic cell	Oxaliplatin encapsulated in cellular vesicles resulted in immunogenic cell death, followed by DC vesicle presentation of tumor antigens to initiate T-cell responses. They also displayed synergistic antitumor effect when combined with anti-PD-L1 therapy.	Mouse model of colon cancer.	(43)
	Erythrocyte vesicles and oncolytic virus	Erythrocyte	Oncolytic viruses were encapsulated into bioengineered cell vesicles to evade antiviral neutralizing antibodies, reduce systemic toxicity and enhance targeting delivery.	Human liver cancer xenograft tumor model.	(70)
	T cell vesicle-coated nanoparticle	T cell	T cell vesicles retained LFA-1, PD-1, TGF-βR and FasL. They actively targeted tumor tissues through LFA-1/ICAM-1 interaction, rescued antitumor effects of CD8+ T cells by blocking PD-1 and TGF-β, and directly induced apoptosis of tumor cells via Fas/FasL axis.	Subcutaneous tumor models of melanoma and lung cancer. Lung metastasis model of melanoma.	(39)
	Neutrophil vesicle-coated drug-loaded nanoparticle	Neutrophil	Carfilzomib-loaded nanoparticles were encapsulated in neutrophil-derived vesicles. Neutrophil vesicles targeted circulating tumor cells and premetastatic lesion through three pairs of interactions including LFA-1/ICAM-1, β1 integrin/VCAM-1, and CD44/L-selectin.	Lung metastasis and premetastatic mouse model of breast cancer.	(42)
	Monocyte vesicle-coated drug-loaded nanoparticle	Monocyte	Doxorubicin-loaded PLGA nanoparticles were coated with monocyte-derived vesicles to achieve tumor targeting through the interaction of α4β1 integrin with VCAM-1.	Human breast cancer xenograft model.	(40)
**Exogenous stimulation**	Granzyme B, PD-1 and TGF-β receptor	T cell	Cellular vesicles derived from activated T cells contained abundant granzyme B, PD-1 and TGF-β receptors and could exert tumoricidal effect as well as prevent T cell exhaustion.	Mouse model of lung cancer.	(13)
	mRNAs of pro-inflammatory cytokines and αPD-L1	Macrophage	Vesicles extruded from M1 macrophages carried high levels of mRNA of pro-inflammatory cytokines such as IL-6 and TNF-α. They could promote the polarization of macrophages toward M1 type and enhance antitumor efficacy of anti-PD-L1 therapy.	Mouse model of colon cancer. Recurrence and metastasis model of breast cancer and melanoma.	(11, 14)

Cellular vesicles hold great therapeutic promise as drug vehicles for combinational cancer immunotherapy, but translating these concepts into practical treatment approaches has proven challenging. Firstly, a key consideration of choosing cellular vesicles as a biomimetic cancer immunotherapy drug carrier is the low immunogenicity, but this also puts forward a requirement that donor cells have to be highly compatible with the recipient to avoid host rejection response. In the meantime, cellular vesicles homing to tumor lesions rely on homotypic recognition and targeting of tumor cells (28). The requirement for autologous cells, especially autologous tumor cells, limits their transformation to clinical applications. Currently, there are only two clinical trials using tumor cell membrane-derived vesicles for the treatment of malignant pleural effusion (NCT01854866 and NCT02657460) (85). Secondly, the hydrodynamic diameter of proteins is usually around 10 nm, while the diameter of the prepared cellular vesicles is approximately 100 to 200 nm. Therefore, the protein density and topology may need to be considered when overexpressing proteins of interest on the vesicle surface to preserve their biological activity as much as possible. Thirdly, when preparing multifunctional cellular vesicles by hybridization, it was noted that the ratio of two different membranes would affect the function of hybridized vesicles (69). In the case of mixed erythrocyte and tumor cell membrane-derived vesicles, an increase in the proportion of erythrocyte membranes displayed prolonged circulation, while an increase in the proportion of tumor cell membranes improved homotypic targeting ability (69). However, this was difficult to control as precisely as mixed liposome preparation and relied on empirical studies on most occasions. Besides, cellular vesicles are prone to spontaneous aggregation in external solutions. The extracted cell membranes can be stored at ultra-low temperature for a long time but not for extruded cellular vesicles. And there are many other problems to be solved in terms of manufacturing, storage, stability and efficiency.

In conclusion, cellular vesicles inherit the cell membrane and part of cytoplasmic components and functions of parental cells. They can serve as ideal drug vehicles for cancer combinational immunotherapy. Future research is particularly needed in the areas of engineering strategies, long-term stability and *in vivo* fate.

## Author Contributions

CX drafted the paper and prepared the table and figures. XZ and DJ conceived and proofread the manuscript. All authors contributed to the article and approved the submitted version.

## Funding

This study was supported by Shanghai Sailing Program (21YF1401900), National Natural Science Foundation of China (82073752, 81773620, and 81803529), Scientific and Innovative Action Plan of Shanghai (20S11904700 and 20JC1411000).

## Conflict of Interest

The authors declare that the research was conducted in the absence of any commercial or financial relationships that could be construed as a potential conflict of interest.

## Publisher’s Note

All claims expressed in this article are solely those of the authors and do not necessarily represent those of their affiliated organizations, or those of the publisher, the editors and the reviewers. Any product that may be evaluated in this article, or claim that may be made by its manufacturer, is not guaranteed or endorsed by the publisher.
